# Preoperative Peripheral Blood Count in Breast Carcinoma: Predictor of Prognosis or a Routine Test

**DOI:** 10.1155/2015/964392

**Published:** 2015-11-30

**Authors:** Amrit Pal Singh Rana, Manjit Kaur, B. Zonunsanga, Arun Puri, Amarjit Singh Kuka

**Affiliations:** ^1^Department of Surgery, GGS Medical College, Faridkot 151203, India; ^2^Department of Pathology, GGS Medical College, Faridkot 151203, India

## Abstract

*Background.* Peripheral blood count is the first investigation to be done in every patient before surgery. As strong relationship exists between cancer and immune response of the body, clinical stage at presentation and altered hematological parameters can influence the progression of cancer and vice versa.* Settings and Design.* It is a case control study of total 50 cases (35 cases of carcinoma breast and 15 cases of benign breast disease).* Methods.* A case control study was carried out; 35 cases of breast cancer patients were taken prior to surgery and chemotherapy with 15 cases of benign breast disease as control. Clinical staging according to the tumor, node, and metastasis classification (TNMc) was done and was correlated with complete blood count (CBC).* Results.* All the cancer patients were females with overall mean age of 47.96 ± 13.84 years. Amongst all altered blood parameters, correlation of absolute lymphocytic count (*p* value 0.001) with TNMc staging was found significant. Particularly, decrease in absolute leucocytic count was observed with increase in stage of breast carcinoma.* Conclusions*. The stage-specific mean values of absolute lymphocytic counts of preoperative breast cancer patients can be used as an economical tool to know the evolution of disease.

## 1. Introduction

Complete blood count especially lymphocytic count reflects the response of cellular immunity in a cancer patient. Any alteration in hematological parameters influences the disease progression. Depending upon the clinical presentation (TNMc staging), response of various hematological parameters has been correlated and studied. Hemoglobin (Hb) and packed cell volume (PCV) are indirectly associated with increased risk of cardiac failure in cancer patients [1]. Total leucocytic count (TLC), if elevated, predicts poorer prognosis [2]. The prognostic significance of neutrophils, lymphocytes, plasma cells, mean platelet volume (MPV), platelet/lymphocyte ratio, and neutrophil/lymphocyte ratio studied in gastric cancer patients showed influence on overall survival [3].

So it is also important to study complete blood count in breast carcinoma and its correlation with TNMc staging.

## 2. Methods

This was a case control study carried among the breast cancer patients (35 cases) who have undergone breast surgeries at a tertiary health care centre. Patients who had taken chemotherapy were excluded. Breast cancer patients were staged prior to surgery. The patients were staged clinically according to the tumor, node, and metastasis (TNM) classification. Stage I tumor is <2 cm in greatest dimension, with no nodal involvement and no metastasis. Stage II ranges from nonevident tumor to tumor >5 cm in greatest dimension with either no nodal involvement or metastases in movable ipsilateral axillary lymph node(s) but no distant metastases. Stage III tumor size ranges from either not evident to tumor of any size with direct extension to (a) chest wall or (b) skin, with no evidence of regional lymph node metastases or metastases in ipsilateral infraclavicular lymph node(s) with or without axillary lymph node involvement or in clinically apparent ipsilateral internal mammary lymph node(s) and in the presence of clinically evident lymph node metastasis in ipsilateral supraclavicular lymph node(s) with or without axillary or internal mammary lymph node involvement. There is no distant metastasis. Stage IV shows involvement of distant metastases regardless of tumor or nodal status. All patients with a breast mass were carefully examined and the mass was measured. The presence or absence of any signs of local advancement (inflammation, peau d'orange, ulceration, satellite nodules, direct chest wall involvement, mobile or fixed axillary nodes, and supraclavicular lymph node) was noted and recorded. Abdomen and spine were also examined for evidence of spread. CBC records were reevaluated for Hb, PCV, mean corpuscular volume (MCV), mean corpuscular hemoglobin (MCH), and mean corpuscular hemoglobin concentration (MCHC), TLC, differential leucocytic count (DLC) with absolute counts, and platelet parameters and were compared with CBC records of 15 cases of benign breast disease.

## 3. Statistics

The results of 35 histologically diagnosed, prechemotherapy breast cancer patients were analyzed. Patients already on chemotherapy before presentation were excluded from the study. Data were analyzed using SPSS version 16.0. The descriptive data were given as means ± SD. The Pearson chi-square test and analysis of variance were used for the analytic assessment and the differences were considered to be statistically significant and very significant when the *p* value obtained was <0.05, <0.002, respectively. Results were compared with findings of 15 samples of benign breast lesions.

## 4. Results

A total of 50 cases of breast disease were studied. Out of them, 35 cases of breast carcinoma were analyzed. All the patients were females with overall mean age of 47.96 ± 13.84 years (Table 1).

A total of 60.0% of the cases had Hb < 12 mg% at presentation. Using PCV > 38% as the cut-off, 62.9% of cases were anaemic, while 37.1% had normal PCV. The minimum PCV for cases was 16.7%, maximum was 45.2%, and the mean was 35.87 ± 6.0%. The PCV was found not to be significantly associated with staging (*p* = 0.54). Also no significant correlation between PCV of the patients and the staging was noted on analysis of variance. Hb, RBC, and PCV parameters of all the cases in the context of carcinoma staging and benign group are given in Table 2.

Red cell indices for different stages of carcinoma are given in Table 3. The overall mean MCV, MCH, and MCHC of cases of carcinoma were 89.98 ± 5.29 fl, 26.8 ± 0.90 g/L, and 31.64 ± 0.84 g/L, respectively, with no significant correlation with staging (*p* value 0.99, *p* value 0.62, *p* value 0.95, and *p* value 0.96, resp.).

While the mean WBC counts (TLC), absolute neutrophil count (N), absolute lymphocytic count (L), absolute monocytic count (M), and absolute eosinophilic count (E) of cases were 7.57 ± 1.0, 4.56 ± 8.8, 2.49 ± 1.2, 2.9 ± 1.4, and 1.34 ± 7.7, respectively (Table 4), the mean platelet count was 2.61 ± 0.4 (Table 5). Very significant correlation of total leucocytic count (*p* value 0.002) and lymphocytic count (*p* value 0.001) was noted. Significant correlation was observed in case of monocytes (*p* value 0.008).

Other parameters have not shown any significant correlation.

Benign group showed significant correlation of lymphocytic count with clinical staging of carcinoma patients (*p* value = 0). Other parameters showed no significant correlation.

## 5. Discussion

Breast cancer is the second most common carcinoma among females with 99% female prevalence. In the present study conducted, cases considered for analysis were found to be of females only. As evidenced in a study conducted in the past, only one percent of breast cancer develops in males. However, in a study done in Nigeria, 2.0% prevalence of breast cancer was reported amongst male population. Although prevalence of breast carcinoma is different in both males and females, prognosis is similar [4–6].

Breast carcinoma is the leading cause of death in women with more than 1,00,000 cases occurring annually [7]. With the advent of many new techniques, prognosis as well as progression of cancer can be estimated. As routine blood investigations are part and parcel of follow-up of carcinoma patients as well as first investigation being done prior to surgery for carcinoma, it can help in the assessment of disease progression. So it is important to make out that a complete blood cell count (CBC) being low cost, standardized, routinely used tests can still offer useful information regarding the behavior of different malignancies. In the current study no significant correlation between anaemia and breast cancer could be identified. In patients with cancer, >60% cases have Hb < 12 gm% along with altered red cell indices; amongst that 4.7% of the cases had Hb < 7 gm%. Anorexia associated with cancers generally can result in nutritional anaemia seen in the cases. On the other hand metastasis to the bone marrow from breast cancer can be associated with suppression of erythropoiesis. Benign group also have shown anaemia (mean Hb < 10 gm%) probably due to lower socioeconomic status and poor nutrition [5, 8].

The mean TLC was decreased with progression of disease from stage 1 to stage 4. It was in contrast with the Blue Mountains Eye Study cohort of older Australians, where an elevated WBC count was seen with cancer mortality, independent of smoking, diabetes mellitus, fasting glucose levels, and other related factors [9]. However, few prospective studies have examined the putative association between systemic markers of inflammation and incident cancer [2, 10]. However, absolute neutrophil counts of cases have not shown any statistical significance in comparison with staging. But, in cases of stage 1, in comparison with control group and other stages, low counts were observed. The various studies done in the past have emphasized that the patients with an absolute granulocyte count of 6000/mm^3^ or more have a shorter survival than the patients with less than 6000/mm^3^. More or less similar phenomenon was observed independently in patients with advanced carcinoma of the colon in another study [11, 12].

In the present study conducted, decrease in mean absolute TPLC was noted with progression of the disease. It was observed that localized tumor in stage 1 has shown lymphocytosis (5.800 ± 36.76) and lymphocytopenia (1.379 ± 0.762) was noted in stage 4. This finding was comparable with reports of Parkin DM and fellows, who observed that the decrease in TPLC was directly proportional to the progress of the disease [7]. In addition, when compared with benign group, stage 1 of breast carcinoma showed significantly raised absolute TPLC and, on the other hand, stage 4 showed significant decreases in absolute TPLC. Many other studies conducted on breast carcinoma patients have shown similar findings. In one study the peripheral lymphocyte count was found to be comparative on the higher side in patients with localized breast carcinoma as compared with evidence of metastasis [13]. The principal mechanism of tumor immunity is killing of tumor cells by CD8+ cytotoxic T-lymphocyte. The natural killer cells are lymphocytes that are capable of destroying tumor cells without prior sensitization. Lee has reported that patients with a higher peripheral blood CD8 count experienced superior survival in a cohort of 113 women with metastatic or recurrent breast cancer. Better clinical outcomes associated with high absolute lymphocyte count have been also reported in gastric and head and neck carcinomas [14, 15]. However, many tumors downregulate expression of class 1 major histocompatibility complex (MHC) molecules as a way of evading immunity. Lymphocyte count may therefore be elevated or depressed. Nemoto et al. and others observed no change in peripheral lymphocyte count in patients of breast carcinoma [16–18].

In a study done in Japan, it has been shown that patients with peripheral blood monocyte count >300/mm^3^ compared to patients with a count <300/mm^3^ have worse 5-year cancer-related survival. But in present study conducted, absolute monocytic count >300/mm^3^ was observed in stage 1, and it was higher than benign group also where it was <300/mm^3^ [19].

The mean platelet count of cases (291.51 ± 103.38) was also higher than that of benign group (222.82 ± 57.62). Preoperative thrombocytosis has been identified as an adverse prognostic indicator in some malignancies [20, 21]. In another study done on patients with esophageal carcinoma, a high platelet count was found to be associated with tumor progression and poor survival [22].

## 6. Conclusion

In summary, breast cancer patients who presented with deranged full blood count pattern were compared with the benign cases. It was observed that stage-specific mean values of absolute lymphocytic counts of breast cancer patients can be employed as a useful guide to assess the progression of disease; however, further work with large sample sized studies is needed in this field.

## Figures and Tables

**Table 1:** Age-wise distribution of cases in different stages of carcinoma and benign group.

Number of cases (total = 50)	Percent of cases (%) within group	Age (mean ± SD)	Minimum age	Maximum age
Carcinoma stage-wise (*n* = 35)				
1	08.5	49.0 ± 2.8	47	51
2	34.2	45.8 ± 11.0	18	60
3	42.8	48.3 ± 14.3	15	75
4	14.2	52.5 ± 18.8	35	80
Benign group (*n* = 15)	30	35.4 ± 12.6	24	58

**Table 2:** Hb, RBC, and PCV parameters in relation to four stages of carcinoma and benign group.

Hb, RBC, and PCV parameters	Carcinoma stage				Benign group
	1	2	3	4	
Hb (mean ± SD)	12.6 ± 1.48	10.4 ± 2.1	11.6 ± 1.35	12.2 ± 1.66	10.5 ± 1.65
Minimum Hb	11.6	4.9	9.0	10.1	8.2
Maximum Hb	13.7	13.5	14.1	14.3	13.2

RBC (mean ± SD)	4.45 ± 0.02	4.16 ± 0.88	4.3 ± 0.44	4.5 ± 0.55	4.5 ± 0.62
Minimum RBC	4.5	2.57	5.36	3.89	3.3
Maximum RBC	4.6	5.97	3.36	5.31	5.6

PCV (mean ± SD)	42.8 ± 3.39	33.6 ± 7.7	35.9 ± 3.6	38.8 ± 3.8	35.0 ± 5.5
Minimum PCV	40.4	16.7	27.0	33.1	27.2
Maximum PCV	45.2	42.7	42.4	43.5	44.0

**Table 3:** Red cell indices in relation to four stages of carcinoma and benign group.

Red cell indices parameters	Carcinoma stage				Benign group
	1	2	3	4	
MCV (mean ± SD)	93.6 ± 8.2	82.8 ± 11.7	84.2 ± 7.5	85.4 ± 2.5	79.5 ± 8.1
Minimum MCV	87.8	62.1	65.6	81.9	63.0
Maximum MCV	99.4	100.3	95.0	89.0	88.9

MCH (mean ± SD)	27.6 ± 3.11	26.2 ± 4.3	26.78 ± 2.1	26.0 ± 0.7	26.2 ± 4.2
Minimum MCH	25.4	18.3	22.0	26.0	18.0
Maximum MCH	29.8	32.5	30.9	28.1	30.3

MCHC (mean ± SD)	31.1 ± 3.95	30.5 ± 1.8	32.1 ± 2.1	31.8 ± 1.0	31.8 ± 1.9
Minimum MCHC	28.3	26	25.0	30.5	28.0
Maximum MCHC	33.9	32.6	34.1	32.9	34.0

**Table 4:** Relation of various leucocytic parameters with four stages of carcinoma and benign group.

Leucocytic parameters	Carcinoma stage				Benign group
	1	2	3	4	
TLC (mean ± SD)	9.100 ± 2.998	8.619 ± 2.930	7.404 ± 1.686	6.222 ± 1.580	8.543 ± 2.400
Minimum TLC	7.000	16.560	10.970	3.550	4.900
Maximum TLC	11.240	5.200	4.630	7.670	14.200

N (mean ± SD)	3.800 ± 0.325	5.829 ± 2.907	4.614 ± 1.209	4.454 ± 1.098	5.380 ± 1.760
Minimum N	3.600	2.180	7.680	2.730	3.000
Maximum N	4.060	14.170	2.850	5.500	9.200

L (mean ± SD)	5.800 ± 3.676	2.289 ± 1.024	2.127 ± 0.641	1.379 ± 0.762	2.753 ± 0.656
Minimum L	3.200	1.100	0.840	0.500	1.600
Maximum L	8.400	4.200	3.500	2.277	3.800

M (mean ± SD)	0.33 ± 0.25	0.33 ± 0.26	0.30 ± 0.23	0.20 ± 0.05	0.226 ± 0.209
Minimum M	0.150	0.100	0.010	0.138	0.010
Maximum M	0.510	1.106	1.106	0.260	0.650

E (mean ± SD)	0.06 ± 0.04	0.12 ± 0.07	0.21 ± 0.15	0.10 ± 0.03	0.066 ± 0.066
Minimum E	0.030	0.000	0.000	0.040	0.008
Maximum E	0.100	0.250	0.550	0.138	0.200

**Table 5:** Relation of platelets parameters with four stages of carcinoma and benign group.

Platelets parameters	Carcinoma stage				Benign group
	1	2	3	4	
Platelets (mean ± SD)	1.91 ± 1.32	2.87 ± 0.73	2.59 ± 0.88	2.65 ± 1.1	2.63 ± 0.54
Minimum platelets	0.97	1.2	1.36	1.58	1.57
Maximum platelets	2.85	3.75	4.81	4.65	3.34
